# Crystal structures of (*E*)-5-(4-methyl­phen­yl)-1-(pyridin-2-yl)pent-2-en-4-yn-1-one and [3,4-bis(phenyl­ethyn­yl)cyclo­butane-1,2-di­yl]bis­(pyridin-2-yl­methanone)

**DOI:** 10.1107/S2056989020000055

**Published:** 2020-01-14

**Authors:** Ivan E. Ushakov, Ivan S. Odin, Pavel A. Gloukhov, Alexander A. Golovanov, Pavel V. Dorovatovskii, Anna V. Vologzhanina

**Affiliations:** a A.N. Nesmeyanov Institute of Organoelement Compounds of RAS, 28, Vavilova Str., Moscow, 119991, Russian Federation; b Togliatti State University; 14 Belorusskaya Str., Togliatti, 445667, Russian Federation; c National Research Center ’Kurchatov Institute’, pl. Akad. Kurchatova, 1, Moscow, 123098, Russian Federation

**Keywords:** vinyl ketones with acetyl­ene fragment, cyclo­butane derivatives, photoreaction, crystal structure

## Abstract

Recrystallization of (*E*)-5-phenyl-1-(pyridin-2-yl)pent-2-en-4-yn-1-one at room temperature from ethyl­ene glycol in daylight afforded [3,4-bis­(phenyl­ethyn­yl)cyclo­butane-1,2-di­yl)bis­(pyridin-2-yl­methanone], C_32_H_22_N_2_O_2_ (**3**), while (*E*)-5-(4-methyl­phen­yl)-1-(pyridin-2-yl)pent-2-en-4-yn-1-one, C_17_H_13_NO (**2**), remained photoinert. This is the first experimental evidence that pentenynones can be photoreactive when fixed in nearly coplanar parallel positions. During the photoreaction, the bond lengths and angles along the pentenyne chain changed significantly, while the disposition of the pyridyl ring towards the keto group was almost unchanged. The cyclo­butane ring adopts an *rctt* conformation.

## Abstract 2

Upon recrystallization from ethyl­ene glycol in daylight, (*E*)-5-phenyl-1-(pyridin-2-yl)pent-2-en-4-yn-1-one underwent spontaneous [2 + 2] cyclo­addition reaction while (*E*)-5-(4-methyl­phen­yl)-1-(pyridin-2-yl)pent-2-en-4-yn-1-one remained photoinert.

## Chemical context   

**Figure 1:**
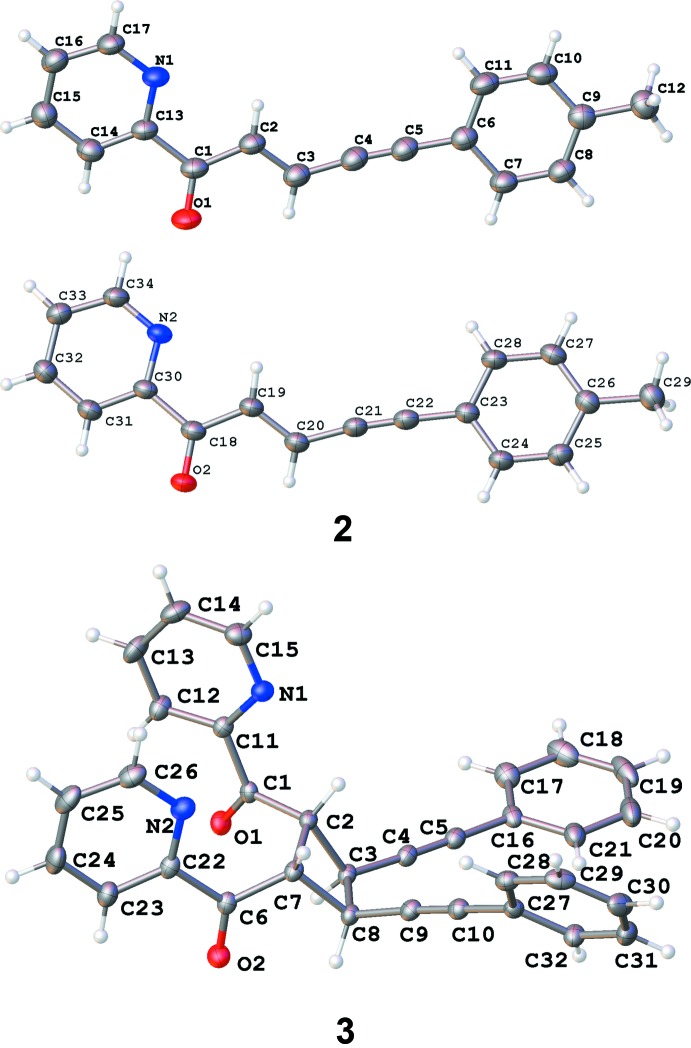
The mol­ecular structure of **2** and **3**, showing the atom-labelling scheme. Displacement ellipsoids are drawn at the 50% probability level.

Vinyl-substituted ketones are known to take part in photo-initiated reactions both in the solid state and in solution (Hopkin *et al.*, 1991 ▸; Vatsadze *et al.*, 2006 ▸). Both *trans*–*cis* isomerization and [2 + 2] cyclo­addition reactions can be observed depending on the nature of the substituents on the alkyl chain (Vatsadze *et al.*, 2006 ▸). Many of the compounds previously reported by us, including 1,5-di­aryl­pentenynones (Golovanov *et al.*, 2013 ▸; Vologzhanina *et al.*, 2014 ▸; Voronova *et al.*, 2016 ▸1) and cyclic ketones with vinyl­acetyl­ene fragments (Voronova *et al.*, 2018 ▸) in crystals exhibit coplanar packing with a distance between the olefin fragments of less than 4.2 Å; thus, they satisfy the Schmidt (1971 ▸) criteria for a solid-state [2 + 2] cyclo­addition to occur. However, our numerous attepts to carry out [2 + 2] photo­cyclo­addition in these compounds were unsuccessful. We aimed to synthesize pyridine-substituted representatives of this family in order to fix olefin fragments in photoreactive positions using hydrogen bonding or coordination bonding as described by Nagarathinam *et al.* (2008 ▸). Two novel pyridine-2-yl-containing ketones, **1** and **2** (Scheme[Chem scheme1] and Fig. 1 ▸), were synthesized as described below, and recrystallized from ethanol. Single-crystal XRD data for **2** could only be obtained using synchrotron radiation, while we failed to obtain a crystal structure of **1** using single-crystal or powder X-ray diffraction. Recrystallization of **1** and **2** from ethyl­ene glycol afforded, respectively, a dimerization reaction product, **3**, and the initial solid phase.
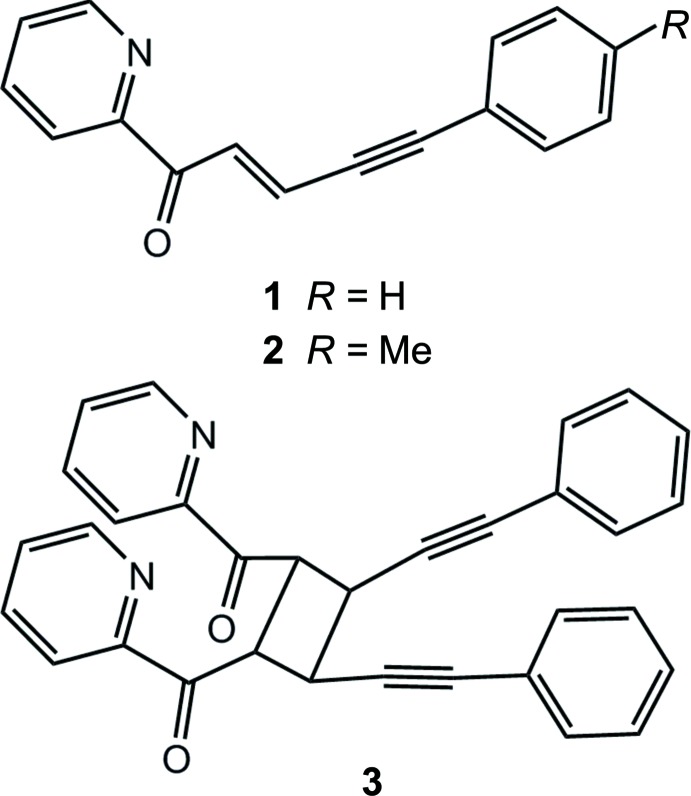


## Structural commentary   

**Figure 2:**
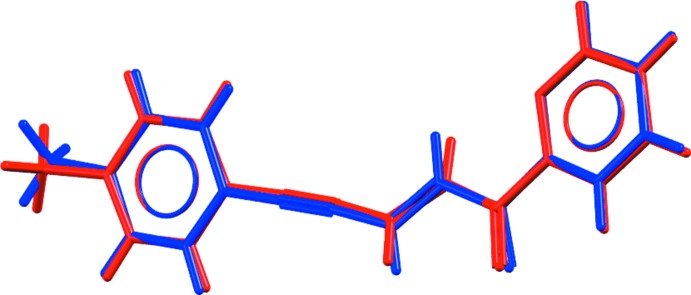
Conformation of the two symmetrically independent mol­ecules in **2** (red and blue) in superimposed representation.

The asymmetric unit of ketone **2** contains two independent mol­ecules (Fig. 1 ▸). Their conformations are very similar to each other as shown in Fig. 2 ▸. Both mol­ecules of **2** exhibit delocalization of charge density along the alkyl chain, as can be concluded from the bond lengths given in Table 1 ▸, the single bonds between a double and a triple bond being much shorter than the average value of 1.53–1.54 Å for a C—C bond. The corresponding values for the C=O ketone fragments in **3** are similar to those in **2**, while the absence of double bonds along the alkyl chain causes shortening of the allyl bonds and elongation of single bonds. The bond lengths in the cyclo­butane ring of **3** are unequal: those corresponding to a previously ‘double’ bond are characteristic of a C—C bond (*ca* 1.55 Å), while the single bonds between two ‘monomers’ are elongated to 1.575 (2) Å. Only the *rctt* isomer of a 1,2,3,4-tetra­substituted cyclo­butane was obtained of four theoretically possible (based on XRD data).

The conformations of the mol­ecules of both **2** and **3** is probably affected by intra­molecular C—H⋯N contacts (Tables 2 ▸ and 3 ▸) involving the nitro­gen atoms of the pyridine-2-yl rings and hydrogen atoms of ethenyl or cyclo­butane moieties. The C—H⋯N angle does not exceed 102°; however, such a mutual disposition of the conjugated pyridine ring and a double bond was found not only in **2** and **3**, but also in previously reported pyridine-2-yl-containing chalcones. The chalcones in the Cambridge Structural Database (CSD, Version 5.40, update of November 2019; Groom *et al.*, 2016 ▸) [ABADUE (Fun *et al.*, 2011*b* ▸), AFOPOC (Chantrapromma *et al.*, 2013 ▸), AYUYOJ (Fun *et al.*, 2011*a* ▸), BERXEC (Wang *et al.*, 2004 ▸), CIBYIY (Brennan *et al.*, 2018 ▸), COBJEJ (Prajapati *et al.*, 2008 ▸), ENINOG (Lee *et al.*, 2016 ▸), GARMAP (Fan & Wang, 2012 ▸), IJUSAI (Jasinski *et al.*, 2011 ▸), IXOXOJ (Dudek *et al.*, 2011 ▸), LANTAY (Qian *et al.*, 2017 ▸), OGIZIP and VUZVET (Tan *et al.*, 2016 ▸), PUKVEY (Rout & Mondal, 2015 ▸), QEMJOK and QEMJUQ (Albaladejo *et al.*, 2018 ▸), SOXHAP (Lin *et al.*, 2009 ▸), TISCEF (Jayarama *et al.*, 2013 ▸) and YUQTEK (Li *et al.*, 2010 ▸)] demonstrate similar conformations, but different crystal packing in the region of pyridyl ring. The majority of 1-phenyl-substituted chalcones and 1-phenyl-substituted pentenyn-1-ones also exhibit a nearly coplanar arrangement of the aryl and ketone fragments and thus no hindrance occurs between the hydrogen atoms of these fragments.

## Supra­molecular features   

**Figure 3:**
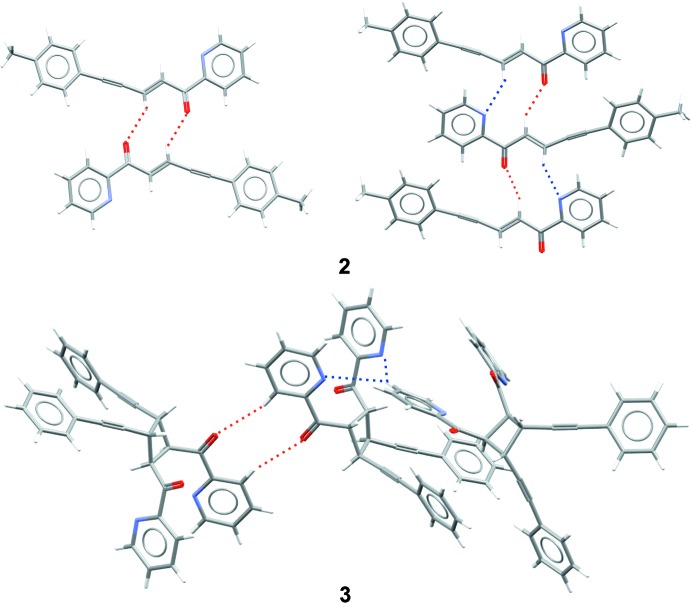
Supra­molecular aggregates in the crystals of **2** and **3**. Hydrogen bonds are depicted by dashed lines.

As the independent mol­ecules of ketone **2** have similar conformations, their crystalline environment becomes of particular inter­est because it can rationalize why *Z* ≠ 1. Previously, we found that the most abundant C—H⋯O-bonded associates in the crystals of chalcones, polyenones and pentenynones include dimers, head-to-tail chains and zigzag C—H⋯O chains with the most acidic proton of a mol­ecule (Vologzhanina *et al.*, 2014 ▸). The two independent mol­ecules of ketone **2** demonstrate two of these motifs (Fig. 3 ▸). In the C—H⋯O-connected dimers, *r*(C⋯O) = 3.206 (3) Å, and in the head-to-tail chains *r*(C⋯O) and *r*(C⋯N) = 3.379 (2) and 3.465 (3) Å, respectively. The corresponding C—H⋯O and C—H⋯N angles are, respectively, 139, 143 and 136°. Note, that only one of two independent mol­ecules in **2** forms head-to-tail chains *via* a pair of inter­molecular C—H⋯O and C—N⋯N bonds. None of the previously reported pyridine-2-yl-containing chalcones nor **3** forms such associates. Instead, the nitro­gen atoms inter­act with the hydrogen atoms of the alkyl and aryl groups. For example, in the crystal of **3**, the hydrogen atoms of a pyridine-2-yl ring take part in C—H⋯N inter­actions [Fig. 3 ▸, *r*(C⋯N) = 3.445 (3)–3.665 (2) Å]. Oxygen atoms take part in C—H⋯O bonding with hydrogen atoms of the phenyl and pyridin-2-yl rings. In addition, in **2** and **3**, numerous hydro­phobic inter­actions can be found.

## Synthesis and crystallization   

The 5-phenyl-1-(pyridin-2-yl)pent-2-en-4-yn-1-one, **1**, and 5-(4-methyl­phen­yl)-1-(pyridin-2-yl)pent-2-en-4-yn-1-one, **2**, were synthesized according to the previously described method (Golovanov *et al.*, 2013 ▸). Single crystals of **3** were grown from solution of **1** in ethyl­ene glycol. The ^1^H NMR spectrum indicates the presence of a mixture of reaction products and unreacted **1**. Powder XRD indicated that the solid sample of the recrystallized ketone consisted of both **1** and **3**, and thus solid **3** could not be characterized by other physicochemical methods. Recrystallization of **2** from ethyl­ene glycol afforded **2** as obtained from XRD data.

For **1**: yellowish needles, yield 61%, m.p. 348–351 K (from a mixture of water and ethanol). ^1^H NMR (300 MHz, CDCl_3_), δ, ppm: 8.48 *s* (1C, C_Ar_, C_Py_), 8.09–8.16 *m* (2C, C_Ar_, C_Py_, C^2^), 7.84–7.79 *m* (2C, C_Ar_, C_Py_), 7.20–7.52 *m* (6C, C_Ar_, C^3^). ^13^C NMR (75 MHz, CDCl_3_), δ, ppm: 188.5 (C1), 152.6, 149.0, 132.2, 131.9, 129.5, 128.6, 128.2, 128.0, 127.1, 122.1, 99.6 (C^5^), 88.9 (C^4^). Found, %: C 82.44; H 5.41. C_16_H_11_NO. Calculated, %: C 82.38; H 4.75.

For **2**: yellowish needles, yield 34%, m.p. 373–374 K (from a mixture of water–ethanol. IR Spectra, ν, cm^−1^: 2191 (C≡C), 1649 (C=O). Found, %: C 82.44; H 5.33. C_17_H_13_NO. Calculated, %: C 82.57; H 5.30.

## Refinement   

Crystal data, data collection and structure refinement details are summarized in Table 4 ▸. Intensity data for **2** were collected at the K4.4 ‘Belok’ beamline of the Kurchatov Synchrotron Radiation Source (NRC ‘Kurchatov Institute’, Moscow, Russia) at a wavelength of 0.80248 Å using a Rayonix CCD 165 detector. Image integration was performed using *iMosflm* software (Battye *et al.*, 2011 ▸). Hydrogen atoms were placed in calculated positions (0.95–1.00 Å) and refined using a riding model, with *U*_iso_(H) = 1.2*U*_eq_(C).

## Supplementary Material

Crystal structure: contains datablock(s) global, 2, 3. DOI: 10.1107/S2056989020000055/ff2164sup1.cif

Structure factors: contains datablock(s) 3. DOI: 10.1107/S2056989020000055/ff21643sup2.hkl

Supporting information file. DOI: 10.1107/S2056989020000055/ff21643sup4.cml

Structure factors: contains datablock(s) 2. DOI: 10.1107/S2056989020000055/ff21642sup3.hkl

Supporting information file. DOI: 10.1107/S2056989020000055/ff21642sup5.cml

CCDC references: 1975319, 1975318

Additional supporting information:  crystallographic information; 3D view; checkCIF report

## Figures and Tables

**Table 1:** Selected geometry parameters (Å, °) for **2** and **3** The carbon atoms of the pentenynone fragment are numbered from 1 to 5. Φ_1_ is the dihedral angle between the pyridine ring and the ketone fragment and Φ_2_ is the dihedral angle between the pyridine and phenyl rings.

Bond	**2**	**3**
C1=O	1.226 (2), 1.228 (2)	1.212 (2), 1.215 (2)
C1—C_py_	1.498 (3)–1.498 (2)	1.495 (2), 1.498 (2)
C1—C2	1.474 (3)–1.477 (3)	1.509 (2), 1.513 (2)
C2=C3	1.335 (3), 1.336 (3)	–
C_cb_—C_cb_	–	1.549 (2), 1.554 (2)
C3—C4	1.411 (3), 1.420 (3)	1.454 (2), 1.460 (2)
C4≡C5	1.206 (3), 1.203 (3)	1.195 (2), 1.194 (2)
C5—C_Ph_	1.426 (3), 1.430 (3)	1.441 (2), 1.439 (2)
Φ_1_	11.0 (1), 11.1 (1)	14.8 (1), 0.9 (1)
Φ_2_	7.4 (1), 5.1 (1)	84.8 (1), 47.0 (1)

**Table 2:** Hydrogen-bond geometry (Å, °) for (2)

*D*—H⋯*A*	*D*—H	H⋯*A*	*D*⋯*A*	*D*—H⋯*A*
C2—H2⋯N1	0.95	2.52	2.832 (3)	100
C16—H16⋯N2^i^	0.95	2.66	3.555 (3)	158
C20—H20⋯N2^i^	0.95	2.71	3.465 (3)	136
C3—H3⋯O1^ii^	0.95	2.43	3.206 (3)	139
C19—H19⋯O2^iii^	0.95	2.57	3.379 (2)	143
C25—H25⋯O2^iv^	0.95	2.65	3.561 (2)	161

Symmetry codes: (i) 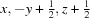; (ii) 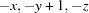; (iii) 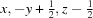; (iv) 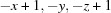.

**Table 3:** Hydrogen-bond geometry (Å, °) for (3)

*D*—H⋯*A*	*D*—H	H⋯*A*	*D*⋯*A*	*D*—H⋯*A*
C2—H2⋯N1	1.00	2.45	2.835 (3)	102
C3—H3⋯O1	1.00	2.49	2.899 (2)	104
C8—H8⋯O2	1.00	2.39	2.804 (2)	104
C25—H25⋯N1^i^	0.95	2.60	3.445 (3)	148
C19—H19⋯N1^ii^	0.95	2.73	3.665 (2)	167
C20—H20⋯O1^iii^	0.95	2.62	3.263 (2)	125
C32—H32⋯O2^iv^	0.95	2.55	3.487 (2)	168

Symmetry codes: (i) 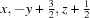; (ii) 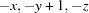; (iii) 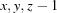; (iv) 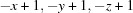.

**Table 4:** Experimental details

	(3)	(2)
Crystal data		
Chemical formula	C_32_H_22_N_2_O_2_	C_17_H_13_NO
*M*_r_	466.51	247.28
Crystal system, space group	Monoclinic, *P*2_1_/*c*	Monoclinic, *P*2_1_/*c*
Temperature (K)	120	100
*a*, *b*, *c* (Å)	12.272 (3), 18.720 (4), 11.425 (2)	14.859 (3), 17.747 (4), 9.995 (2)
β (°)	115.850 (3)	101.06 (3)
*V* (Å^3^)	2362.0 (8)	2586.7 (9)
*Z*	4	8
Radiation type	Mo *K*α	Synchrotron, λ = 0.80248 Å
μ (mm^−1^)	0.08	0.10
Crystal size (mm)	0.46 × 0.28 × 0.17	0.02 × 0.02 × 0.01

Data collection		
Diffractometer	Bruker SMART APEX CCD area detector	Mar CCD
Absorption correction	Multi-scan (*SADABS*; Bruker, 2014 ▸)	Multi-scan (*SCALA*; Evans, 2006 ▸)
*T*_min_, *T*_max_	0.848, 0.903	0.997, 0.999
No. of measured, independent and observed [*I* > 2σ(*I*)] reflections	24543, 7095, 4255	23024, 5645, 4453
*R*_int_	0.079	0.077
(sin θ/λ)_max_ (Å^−1^)	0.714	0.640

Refinement		
*R*[*F* ^2^ > 2σ(*F* ^2^)], *wR*(*F* ^2^), *S*	0.056, 0.130, 0.99	0.060, 0.157, 1.02
No. of reflections	7095	5645
No. of parameters	325	346
H-atom treatment	H-atom parameters constrained	H-atom parameters constrained
Δρ_max_, Δρ_min_ (e Å^−3^)	0.33, −0.31	0.23, −0.21

Computer programs: *APEX2* and *SAINT* (Bruker, 2014 ▸), *Marccd* (Doyle, 2011 ▸), *iMosflm* (Battye *et al.*, 2011 ▸), *SHELXT* (Sheldrick, 2015*a* ▸), *SHELXL* (Sheldrick, 2015*b* ▸) and *OLEX2* (Dolomanov *et al.*, 2009 ▸).

## supplementary crystallographic information

### [3,4-Bis(phenylethynyl)cyclobutane-1,2-diyl]bis(pyridin-2-ylmethanone) (3) Crystal data

**Table:**

C_32_H_22_N_2_O_2_	*F*(000) = 976
*M**_r_* = 466.51	*D*_x_ = 1.312 Mg m^−^^3^
Monoclinic, *P*2_1_/*c*	Mo *K*α radiation, λ = 0.71073 Å
*a* = 12.272 (3) Å	Cell parameters from 4566 reflections
*b* = 18.720 (4) Å	θ = 2.2–30.0°
*c* = 11.425 (2) Å	µ = 0.08 mm^−^^1^
β = 115.850 (3)°	*T* = 120 K
*V* = 2362.0 (8) Å^3^	Prism, orange
*Z* = 4	0.46 × 0.28 × 0.17 mm

### [3,4-Bis(phenylethynyl)cyclobutane-1,2-diyl]bis(pyridin-2-ylmethanone) (3) Data collection

**Table:**

Bruker SMART APEX CCD area detector diffractometer	7095 independent reflections
Radiation source: sealed tube	4255 reflections with *I* > 2σ(*I*)
Graphite monochromator	*R*_int_ = 0.079
Detector resolution: 8 pixels mm^-1^	θ_max_ = 30.5°, θ_min_ = 1.8°
ω scans	*h* = −15→17
Absorption correction: multi-scan (SADABS; Bruker, 2014)	*k* = −26→26
*T*_min_ = 0.848, *T*_max_ = 0.903	*l* = −16→16
24543 measured reflections	

### [3,4-Bis(phenylethynyl)cyclobutane-1,2-diyl]bis(pyridin-2-ylmethanone) (3) Refinement

**Table:**

Refinement on *F*^2^	Primary atom site location: dual
Least-squares matrix: full	Hydrogen site location: inferred from neighbouring sites
*R*[*F*^2^ > 2σ(*F*^2^)] = 0.056	H-atom parameters constrained
*wR*(*F*^2^) = 0.130	*w* = 1/[σ^2^(*F*_o_^2^) + (0.0557*P*)^2^] where *P* = (*F*_o_^2^ + 2*F*_c_^2^)/3
*S* = 0.99	(Δ/σ)_max_ < 0.001
7095 reflections	Δρ_max_ = 0.33 e Å^−^^3^
325 parameters	Δρ_min_ = −0.31 e Å^−^^3^
0 restraints	

### [3,4-Bis(phenylethynyl)cyclobutane-1,2-diyl]bis(pyridin-2-ylmethanone) (3) Special details

**Table:**

Geometry. All esds (except the esd in the dihedral angle between two l.s. planes) are estimated using the full covariance matrix. The cell esds are taken into account individually in the estimation of esds in distances, angles and torsion angles; correlations between esds in cell parameters are only used when they are defined by crystal symmetry. An approximate (isotropic) treatment of cell esds is used for estimating esds involving l.s. planes.

### [3,4-Bis(phenylethynyl)cyclobutane-1,2-diyl]bis(pyridin-2-ylmethanone) (3) Fractional atomic coordinates and isotropic or equivalent isotropic displacement parameters (Å^2^)

**Table:**

	*x*	*y*	*z*	*U*_iso_*/*U*_eq_	
O1	0.14655 (11)	0.50117 (6)	0.61894 (10)	0.0224 (3)	
O2	0.44626 (12)	0.53397 (6)	0.81027 (11)	0.0249 (3)	
N1	0.03170 (13)	0.66755 (7)	0.47517 (13)	0.0215 (3)	
N2	0.26664 (14)	0.68759 (7)	0.76527 (14)	0.0236 (3)	
C1	0.14015 (15)	0.55793 (8)	0.56522 (14)	0.0167 (3)	
C2	0.23152 (15)	0.57818 (8)	0.51555 (15)	0.0168 (3)	
H2	0.1954	0.6114	0.4396	0.020*	
C3	0.30105 (15)	0.51652 (8)	0.48769 (15)	0.0173 (3)	
H3	0.3031	0.4743	0.5421	0.021*	
C4	0.25950 (16)	0.49385 (8)	0.35333 (15)	0.0188 (3)	
C5	0.22108 (16)	0.47470 (8)	0.24269 (16)	0.0202 (4)	
C6	0.38544 (15)	0.58614 (8)	0.75844 (15)	0.0177 (3)	
C7	0.35663 (15)	0.60721 (8)	0.62014 (15)	0.0175 (3)	
H7	0.3676	0.6595	0.6106	0.021*	
C8	0.42148 (16)	0.55980 (8)	0.55808 (15)	0.0179 (3)	
H8	0.4875	0.5304	0.6245	0.021*	
C9	0.46337 (16)	0.59998 (8)	0.47593 (15)	0.0198 (3)	
C10	0.50391 (16)	0.63388 (8)	0.41551 (15)	0.0200 (4)	
C11	0.04630 (15)	0.61189 (8)	0.55421 (15)	0.0171 (3)	
C12	−0.01798 (17)	0.60341 (8)	0.62740 (17)	0.0241 (4)	
H12	−0.0064	0.5624	0.6805	0.029*	
C13	−0.09935 (18)	0.65600 (9)	0.62137 (19)	0.0303 (4)	
H13	−0.1441	0.6521	0.6711	0.036*	
C14	−0.11435 (18)	0.71411 (9)	0.54191 (18)	0.0302 (4)	
H14	−0.1687	0.7514	0.5367	0.036*	
C15	−0.04852 (17)	0.71700 (9)	0.46982 (17)	0.0270 (4)	
H15	−0.0612	0.7565	0.4132	0.032*	
C16	0.17317 (16)	0.45234 (8)	0.10882 (15)	0.0208 (4)	
C17	0.06057 (18)	0.42003 (8)	0.04773 (17)	0.0282 (4)	
H17	0.0149	0.4108	0.0952	0.034*	
C18	0.0143 (2)	0.40106 (9)	−0.08242 (17)	0.0364 (5)	
H18	−0.0629	0.3789	−0.1238	0.044*	
C19	0.0804 (2)	0.41430 (10)	−0.15217 (18)	0.0406 (6)	
H19	0.0486	0.4013	−0.2413	0.049*	
C20	0.1925 (2)	0.44636 (10)	−0.09211 (18)	0.0370 (5)	
H20	0.2375	0.4558	−0.1402	0.044*	
C21	0.23975 (19)	0.46490 (9)	0.03804 (16)	0.0274 (4)	
H21	0.3177	0.4862	0.0794	0.033*	
C22	0.33415 (15)	0.63164 (8)	0.83025 (15)	0.0187 (3)	
C23	0.35862 (17)	0.61505 (9)	0.95735 (16)	0.0244 (4)	
H23	0.4081	0.5753	1.0000	0.029*	
C24	0.30935 (18)	0.65777 (10)	1.02106 (18)	0.0313 (4)	
H24	0.3238	0.6477	1.1081	0.038*	
C25	0.23927 (18)	0.71490 (9)	0.95603 (18)	0.0309 (4)	
H25	0.2044	0.7452	0.9972	0.037*	
C26	0.22022 (18)	0.72774 (9)	0.82891 (18)	0.0290 (4)	
H26	0.1713	0.7674	0.7847	0.035*	
C27	0.55587 (15)	0.67731 (8)	0.34887 (15)	0.0187 (3)	
C28	0.56084 (16)	0.75148 (8)	0.36530 (16)	0.0224 (4)	
H28	0.5309	0.7727	0.4210	0.027*	
C29	0.60909 (17)	0.79383 (9)	0.30078 (18)	0.0279 (4)	
H29	0.6126	0.8442	0.3126	0.033*	
C30	0.65229 (18)	0.76346 (9)	0.21905 (18)	0.0292 (4)	
H30	0.6837	0.7930	0.1733	0.035*	
C31	0.64973 (18)	0.69037 (9)	0.20388 (17)	0.0289 (4)	
H31	0.6810	0.6696	0.1490	0.035*	
C32	0.60178 (17)	0.64693 (8)	0.26814 (16)	0.0227 (4)	
H32	0.6002	0.5966	0.2572	0.027*	

### [3,4-Bis(phenylethynyl)cyclobutane-1,2-diyl]bis(pyridin-2-ylmethanone) (3) Atomic displacement parameters (Å^2^)

**Table:**

	*U*^11^	*U*^22^	*U*^33^	*U*^12^	*U*^13^	*U*^23^
O1	0.0294 (8)	0.0176 (5)	0.0243 (6)	0.0001 (5)	0.0154 (6)	0.0028 (4)
O2	0.0310 (8)	0.0202 (6)	0.0250 (6)	0.0042 (5)	0.0136 (6)	0.0002 (5)
N1	0.0219 (9)	0.0185 (7)	0.0245 (7)	0.0011 (6)	0.0103 (7)	0.0008 (5)
N2	0.0242 (9)	0.0190 (7)	0.0269 (7)	−0.0005 (6)	0.0105 (7)	−0.0052 (6)
C1	0.0200 (10)	0.0153 (7)	0.0154 (7)	−0.0033 (6)	0.0084 (7)	−0.0035 (6)
C2	0.0211 (10)	0.0136 (7)	0.0182 (7)	0.0017 (6)	0.0109 (7)	0.0010 (6)
C3	0.0228 (10)	0.0126 (7)	0.0203 (8)	0.0007 (6)	0.0130 (7)	−0.0006 (6)
C4	0.0223 (10)	0.0140 (7)	0.0239 (8)	−0.0001 (6)	0.0136 (7)	0.0006 (6)
C5	0.0233 (10)	0.0159 (7)	0.0245 (8)	0.0015 (6)	0.0133 (8)	0.0007 (6)
C6	0.0206 (10)	0.0142 (7)	0.0198 (7)	−0.0042 (6)	0.0102 (7)	−0.0039 (6)
C7	0.0216 (10)	0.0125 (7)	0.0218 (8)	−0.0005 (6)	0.0125 (7)	−0.0015 (6)
C8	0.0218 (10)	0.0150 (7)	0.0205 (8)	0.0005 (6)	0.0126 (7)	0.0000 (6)
C9	0.0215 (10)	0.0164 (7)	0.0231 (8)	0.0004 (6)	0.0114 (7)	−0.0030 (6)
C10	0.0217 (10)	0.0173 (7)	0.0212 (8)	0.0010 (6)	0.0096 (7)	−0.0025 (6)
C11	0.0177 (10)	0.0141 (7)	0.0196 (8)	−0.0034 (6)	0.0083 (7)	−0.0034 (6)
C12	0.0290 (11)	0.0183 (8)	0.0311 (9)	−0.0010 (7)	0.0189 (8)	−0.0010 (7)
C13	0.0284 (12)	0.0279 (9)	0.0449 (11)	−0.0024 (8)	0.0255 (10)	−0.0047 (8)
C14	0.0235 (12)	0.0241 (9)	0.0448 (11)	0.0035 (7)	0.0167 (9)	−0.0025 (8)
C15	0.0268 (12)	0.0201 (8)	0.0324 (10)	0.0024 (7)	0.0115 (8)	0.0041 (7)
C16	0.0316 (12)	0.0130 (7)	0.0191 (8)	0.0045 (6)	0.0122 (8)	0.0008 (6)
C17	0.0356 (13)	0.0203 (8)	0.0275 (9)	−0.0002 (7)	0.0127 (9)	−0.0025 (7)
C18	0.0452 (15)	0.0226 (9)	0.0277 (10)	0.0036 (8)	0.0032 (9)	−0.0061 (7)
C19	0.0704 (18)	0.0258 (10)	0.0180 (9)	0.0156 (10)	0.0121 (10)	−0.0015 (7)
C20	0.0578 (16)	0.0339 (10)	0.0260 (9)	0.0149 (10)	0.0246 (10)	0.0060 (8)
C21	0.0369 (13)	0.0244 (9)	0.0240 (9)	0.0076 (8)	0.0162 (9)	0.0051 (7)
C22	0.0206 (10)	0.0155 (7)	0.0224 (8)	−0.0058 (6)	0.0115 (7)	−0.0071 (6)
C23	0.0282 (12)	0.0238 (8)	0.0251 (9)	−0.0019 (7)	0.0152 (8)	−0.0044 (7)
C24	0.0352 (13)	0.0381 (10)	0.0260 (9)	−0.0031 (8)	0.0184 (9)	−0.0091 (8)
C25	0.0293 (12)	0.0304 (9)	0.0374 (10)	−0.0038 (8)	0.0187 (9)	−0.0169 (8)
C26	0.0275 (12)	0.0219 (8)	0.0378 (10)	0.0016 (7)	0.0144 (9)	−0.0079 (7)
C27	0.0174 (10)	0.0189 (7)	0.0198 (8)	0.0005 (6)	0.0083 (7)	0.0017 (6)
C28	0.0209 (11)	0.0198 (8)	0.0248 (9)	0.0022 (7)	0.0084 (8)	−0.0008 (6)
C29	0.0249 (11)	0.0150 (8)	0.0378 (10)	0.0012 (7)	0.0081 (9)	0.0057 (7)
C30	0.0258 (12)	0.0293 (9)	0.0354 (10)	0.0011 (7)	0.0161 (9)	0.0136 (8)
C31	0.0308 (12)	0.0337 (10)	0.0288 (9)	0.0061 (8)	0.0192 (9)	0.0060 (8)
C32	0.0285 (11)	0.0178 (8)	0.0249 (9)	0.0033 (7)	0.0146 (8)	0.0020 (6)

### [3,4-Bis(phenylethynyl)cyclobutane-1,2-diyl]bis(pyridin-2-ylmethanone) (3) Geometric parameters (Å, º)

**Table:**

O1—C1	1.2123 (18)	C15—H15	0.9500
O2—C6	1.2148 (19)	C16—C17	1.386 (3)
N1—C11	1.3387 (19)	C16—C21	1.397 (2)
N1—C15	1.333 (2)	C17—H17	0.9500
N2—C22	1.341 (2)	C17—C18	1.387 (2)
N2—C26	1.334 (2)	C18—H18	0.9500
C1—C2	1.509 (2)	C18—C19	1.385 (3)
C1—C11	1.495 (2)	C19—H19	0.9500
C2—H2	1.0000	C19—C20	1.378 (3)
C2—C3	1.549 (2)	C20—H20	0.9500
C2—C7	1.575 (2)	C20—C21	1.384 (3)
C3—H3	1.0000	C21—H21	0.9500
C3—C4	1.454 (2)	C22—C23	1.384 (2)
C3—C8	1.566 (2)	C23—H23	0.9500
C4—C5	1.195 (2)	C23—C24	1.385 (2)
C5—C16	1.441 (2)	C24—H24	0.9500
C6—C7	1.513 (2)	C24—C25	1.370 (3)
C6—C22	1.498 (2)	C25—H25	0.9500
C7—H7	1.0000	C25—C26	1.388 (3)
C7—C8	1.554 (2)	C26—H26	0.9500
C8—H8	1.0000	C27—C28	1.399 (2)
C8—C9	1.460 (2)	C27—C32	1.395 (2)
C9—C10	1.194 (2)	C28—H28	0.9500
C10—C27	1.439 (2)	C28—C29	1.380 (2)
C11—C12	1.386 (2)	C29—H29	0.9500
C12—H12	0.9500	C29—C30	1.381 (3)
C12—C13	1.382 (2)	C30—H30	0.9500
C13—H13	0.9500	C30—C31	1.378 (2)
C13—C14	1.377 (3)	C31—H31	0.9500
C14—H14	0.9500	C31—C32	1.387 (2)
C14—C15	1.384 (2)	C32—H32	0.9500
			
C15—N1—C11	116.77 (14)	C17—C16—C5	121.14 (15)
C26—N2—C22	116.48 (15)	C17—C16—C21	119.21 (16)
O1—C1—C2	121.04 (14)	C21—C16—C5	119.63 (17)
O1—C1—C11	120.81 (14)	C16—C17—H17	119.9
C11—C1—C2	118.03 (13)	C16—C17—C18	120.23 (18)
C1—C2—H2	111.2	C18—C17—H17	119.9
C1—C2—C3	117.15 (12)	C17—C18—H18	119.9
C1—C2—C7	116.08 (12)	C19—C18—C17	120.2 (2)
C3—C2—H2	111.2	C19—C18—H18	119.9
C3—C2—C7	88.33 (12)	C18—C19—H19	120.0
C7—C2—H2	111.2	C20—C19—C18	119.94 (17)
C2—C3—H3	109.2	C20—C19—H19	120.0
C2—C3—C8	89.43 (11)	C19—C20—H20	119.9
C4—C3—C2	117.61 (14)	C19—C20—C21	120.24 (19)
C4—C3—H3	109.2	C21—C20—H20	119.9
C4—C3—C8	120.76 (13)	C16—C21—H21	119.9
C8—C3—H3	109.2	C20—C21—C16	120.2 (2)
C5—C4—C3	177.49 (18)	C20—C21—H21	119.9
C4—C5—C16	179.10 (19)	N2—C22—C6	116.52 (14)
O2—C6—C7	122.18 (14)	N2—C22—C23	123.73 (14)
O2—C6—C22	120.37 (14)	C23—C22—C6	119.75 (15)
C22—C6—C7	117.44 (13)	C22—C23—H23	120.7
C2—C7—H7	112.8	C22—C23—C24	118.50 (16)
C6—C7—C2	114.14 (13)	C24—C23—H23	120.7
C6—C7—H7	112.8	C23—C24—H24	120.7
C6—C7—C8	113.24 (13)	C25—C24—C23	118.70 (16)
C8—C7—C2	88.89 (11)	C25—C24—H24	120.7
C8—C7—H7	112.8	C24—C25—H25	120.6
C3—C8—H8	112.1	C24—C25—C26	118.82 (16)
C7—C8—C3	88.48 (12)	C26—C25—H25	120.6
C7—C8—H8	112.1	N2—C26—C25	123.76 (17)
C9—C8—C3	117.03 (13)	N2—C26—H26	118.1
C9—C8—C7	113.14 (12)	C25—C26—H26	118.1
C9—C8—H8	112.1	C28—C27—C10	119.56 (14)
C10—C9—C8	175.96 (18)	C32—C27—C10	121.30 (14)
C9—C10—C27	176.81 (17)	C32—C27—C28	119.14 (15)
N1—C11—C1	117.01 (14)	C27—C28—H28	119.9
N1—C11—C12	123.50 (15)	C29—C28—C27	120.17 (16)
C12—C11—C1	119.46 (14)	C29—C28—H28	119.9
C11—C12—H12	120.8	C28—C29—H29	119.8
C13—C12—C11	118.50 (15)	C28—C29—C30	120.35 (15)
C13—C12—H12	120.8	C30—C29—H29	119.8
C12—C13—H13	120.6	C29—C30—H30	120.0
C14—C13—C12	118.78 (16)	C31—C30—C29	119.96 (16)
C14—C13—H13	120.6	C31—C30—H30	120.0
C13—C14—H14	120.7	C30—C31—H31	119.8
C13—C14—C15	118.58 (16)	C30—C31—C32	120.49 (16)
C15—C14—H14	120.7	C32—C31—H31	119.8
N1—C15—C14	123.84 (16)	C27—C32—H32	120.1
N1—C15—H15	118.1	C31—C32—C27	119.87 (15)
C14—C15—H15	118.1	C31—C32—H32	120.1

### [3,4-Bis(phenylethynyl)cyclobutane-1,2-diyl]bis(pyridin-2-ylmethanone) (3) Hydrogen-bond geometry (Å, º)

**Table:**

*D*—H···*A*	*D*—H	H···*A*	*D*···*A*	*D*—H···*A*
C2—H2···N1	1.00	2.45	2.835 (3)	102
C3—H3···O1	1.00	2.49	2.899 (2)	104
C8—H8···O2	1.00	2.39	2.804 (2)	104
C25—H25···N1^i^	0.95	2.60	3.445 (3)	148
C19—H19···N1^ii^	0.95	2.73	3.665 (2)	167
C20—H20···O1^iii^	0.95	2.62	3.263 (2)	125
C32—H32···O2^iv^	0.95	2.55	3.487 (2)	168

Symmetry codes: (i) *x*, −*y*+3/2, *z*+1/2; (ii) −*x*, −*y*+1, −*z*; (iii) *x*, *y*, *z*−1; (iv) −*x*+1, −*y*+1, −*z*+1.

### (E)-5-(4-Methylphenyl)-1-(pyridin-2-yl)pent-2-en-4-yn-1-one (2) Crystal data

**Table:**

C_17_H_13_NO	*D*_x_ = 1.270 Mg m^−^^3^
*M**_r_* = 247.28	Melting point: 373 K
Monoclinic, *P*2_1_/*c*	Synchrotron radiation, λ = 0.80248 Å
*a* = 14.859 (3) Å	Cell parameters from 148 reflections
*b* = 17.747 (4) Å	θ = 3.5–25.6°
*c* = 9.995 (2) Å	µ = 0.10 mm^−^^1^
β = 101.06 (3)°	*T* = 100 K
*V* = 2586.7 (9) Å^3^	Plate, yellow
*Z* = 8	0.02 × 0.02 × 0.01 mm
*F*(000) = 1040	

### (E)-5-(4-Methylphenyl)-1-(pyridin-2-yl)pent-2-en-4-yn-1-one (2) Data collection

**Table:**

Mar CCD diffractometer	4453 reflections with *I* > 2σ(*I*)
phi scans	*R*_int_ = 0.077
Absorption correction: multi-scan (*SCALA*; Evans, 2006)	θ_max_ = 30.9°, θ_min_ = 3.3°
*T*_min_ = 0.997, *T*_max_ = 0.999	*h* = −18→18
23024 measured reflections	*k* = −22→22
5645 independent reflections	*l* = −12→12

### (E)-5-(4-Methylphenyl)-1-(pyridin-2-yl)pent-2-en-4-yn-1-one (2) Refinement

**Table:**

Refinement on *F*^2^	Hydrogen site location: inferred from neighbouring sites
Least-squares matrix: full	H-atom parameters constrained
*R*[*F*^2^ > 2σ(*F*^2^)] = 0.060	*w* = 1/[σ^2^(*F*_o_^2^) + (0.057*P*)^2^ + 1.189*P*] where *P* = (*F*_o_^2^ + 2*F*_c_^2^)/3
*wR*(*F*^2^) = 0.157	(Δ/σ)_max_ < 0.001
*S* = 1.02	Δρ_max_ = 0.23 e Å^−^^3^
5645 reflections	Δρ_min_ = −0.21 e Å^−^^3^
346 parameters	Extinction correction: SHELXL, Fc^*^=kFc[1+0.001xFc^2^λ^3^/sin(2θ)]^-1/4^
0 restraints	Extinction coefficient: 0.051 (4)

### (E)-5-(4-Methylphenyl)-1-(pyridin-2-yl)pent-2-en-4-yn-1-one (2) Special details

**Table:**

Geometry. All esds (except the esd in the dihedral angle between two l.s. planes) are estimated using the full covariance matrix. The cell esds are taken into account individually in the estimation of esds in distances, angles and torsion angles; correlations between esds in cell parameters are only used when they are defined by crystal symmetry. An approximate (isotropic) treatment of cell esds is used for estimating esds involving l.s. planes.

### (E)-5-(4-Methylphenyl)-1-(pyridin-2-yl)pent-2-en-4-yn-1-one (2) Fractional atomic coordinates and isotropic or equivalent isotropic displacement parameters (Å^2^)

**Table:**

	*x*	*y*	*z*	*U*_iso_*/*U*_eq_	
O1	0.08252 (11)	0.41331 (9)	0.08638 (13)	0.0493 (4)	
N1	0.19600 (11)	0.39239 (9)	0.42804 (15)	0.0383 (4)	
C1	0.11482 (13)	0.43098 (12)	0.20438 (18)	0.0379 (4)	
C2	0.12405 (13)	0.51037 (11)	0.24820 (19)	0.0380 (4)	
H2	0.1567	0.5228	0.3367	0.046*	
C3	0.08643 (14)	0.56483 (12)	0.1634 (2)	0.0409 (5)	
H3	0.0526	0.5493	0.0773	0.049*	
C4	0.09180 (14)	0.64305 (12)	0.1887 (2)	0.0420 (5)	
C5	0.09537 (14)	0.71075 (12)	0.1993 (2)	0.0413 (5)	
C6	0.10213 (14)	0.79075 (12)	0.20949 (19)	0.0394 (4)	
C7	0.07036 (14)	0.83675 (12)	0.0969 (2)	0.0418 (5)	
H7	0.0431	0.8147	0.0122	0.050*	
C8	0.07839 (14)	0.91450 (12)	0.1084 (2)	0.0414 (5)	
H8	0.0561	0.9450	0.0311	0.050*	
C9	0.11833 (14)	0.94863 (12)	0.2305 (2)	0.0412 (5)	
C10	0.14997 (15)	0.90211 (13)	0.3424 (2)	0.0467 (5)	
H10	0.1776	0.9242	0.4269	0.056*	
C11	0.14194 (15)	0.82508 (13)	0.3329 (2)	0.0458 (5)	
H11	0.1636	0.7948	0.4108	0.055*	
C12	0.12884 (16)	1.03274 (12)	0.2434 (2)	0.0494 (5)	
H12A	0.1932	1.0464	0.2476	0.074*	
H12B	0.1094	1.0496	0.3268	0.074*	
H12C	0.0908	1.0571	0.1643	0.074*	
C13	0.14687 (13)	0.37043 (11)	0.30695 (18)	0.0353 (4)	
C14	0.12604 (14)	0.29623 (12)	0.2729 (2)	0.0408 (5)	
H14	0.0903	0.2835	0.1865	0.049*	
C15	0.15839 (15)	0.24046 (12)	0.3676 (2)	0.0449 (5)	
H15	0.1445	0.1889	0.3479	0.054*	
C16	0.21129 (15)	0.26184 (12)	0.4913 (2)	0.0431 (5)	
H16	0.2359	0.2251	0.5575	0.052*	
C17	0.22753 (15)	0.33754 (12)	0.51642 (19)	0.0426 (5)	
H17	0.2634	0.3516	0.6020	0.051*	
O2	0.45764 (10)	0.31523 (8)	0.50791 (12)	0.0395 (3)	
N2	0.35093 (11)	0.37903 (9)	0.18087 (14)	0.0342 (4)	
C18	0.42385 (13)	0.31321 (11)	0.38578 (17)	0.0327 (4)	
C19	0.41012 (13)	0.24154 (10)	0.30956 (17)	0.0331 (4)	
H19	0.3996	0.2414	0.2128	0.040*	
C20	0.41275 (13)	0.17693 (10)	0.37887 (18)	0.0342 (4)	
H20	0.4235	0.1806	0.4755	0.041*	
C21	0.40113 (13)	0.10337 (10)	0.32236 (17)	0.0339 (4)	
C22	0.39020 (13)	0.03862 (11)	0.28721 (17)	0.0343 (4)	
C23	0.37756 (13)	−0.03916 (10)	0.25127 (17)	0.0328 (4)	
C24	0.41627 (13)	−0.09465 (10)	0.34423 (17)	0.0343 (4)	
H24	0.4510	−0.0804	0.4304	0.041*	
C25	0.40418 (13)	−0.17024 (11)	0.31134 (18)	0.0362 (4)	
H25	0.4313	−0.2073	0.3751	0.043*	
C26	0.35279 (13)	−0.19286 (11)	0.18589 (18)	0.0354 (4)	
C27	0.31461 (13)	−0.13723 (11)	0.09343 (18)	0.0369 (4)	
H27	0.2800	−0.1516	0.0072	0.044*	
C28	0.32623 (13)	−0.06173 (11)	0.12495 (17)	0.0354 (4)	
H28	0.2993	−0.0248	0.0607	0.042*	
C29	0.33893 (15)	−0.27549 (11)	0.1537 (2)	0.0435 (5)	
H29A	0.3925	−0.3039	0.2002	0.065*	
H29B	0.3313	−0.2831	0.0551	0.065*	
H29C	0.2840	−0.2933	0.1849	0.065*	
C30	0.39586 (12)	0.38512 (10)	0.31068 (16)	0.0312 (4)	
C31	0.41532 (13)	0.45334 (11)	0.37716 (18)	0.0355 (4)	
H31	0.4450	0.4547	0.4702	0.043*	
C32	0.39093 (14)	0.51947 (11)	0.30610 (19)	0.0386 (4)	
H32	0.4052	0.5671	0.3482	0.046*	
C33	0.34521 (14)	0.51431 (11)	0.17197 (19)	0.0380 (4)	
H33	0.3273	0.5585	0.1200	0.046*	
C34	0.32614 (13)	0.44366 (11)	0.11495 (18)	0.0368 (4)	
H34	0.2935	0.4409	0.0235	0.044*	

### (E)-5-(4-Methylphenyl)-1-(pyridin-2-yl)pent-2-en-4-yn-1-one (2) Atomic displacement parameters (Å^2^)

**Table:**

	*U*^11^	*U*^22^	*U*^33^	*U*^12^	*U*^13^	*U*^23^
O1	0.0589 (9)	0.0542 (9)	0.0279 (7)	0.0043 (7)	−0.0087 (6)	−0.0013 (6)
N1	0.0397 (9)	0.0449 (9)	0.0267 (8)	0.0005 (7)	−0.0026 (6)	−0.0014 (6)
C1	0.0361 (10)	0.0482 (11)	0.0266 (9)	0.0012 (8)	−0.0009 (7)	−0.0001 (7)
C2	0.0393 (10)	0.0440 (11)	0.0280 (9)	0.0010 (8)	−0.0005 (7)	0.0017 (7)
C3	0.0403 (11)	0.0461 (11)	0.0337 (10)	−0.0007 (9)	0.0005 (8)	0.0029 (8)
C4	0.0401 (11)	0.0479 (12)	0.0352 (10)	0.0009 (9)	0.0000 (8)	0.0059 (8)
C5	0.0391 (10)	0.0472 (12)	0.0352 (10)	0.0002 (9)	0.0012 (8)	0.0050 (8)
C6	0.0374 (10)	0.0442 (11)	0.0354 (10)	−0.0003 (8)	0.0037 (8)	0.0025 (8)
C7	0.0420 (11)	0.0485 (12)	0.0317 (9)	−0.0002 (9)	−0.0015 (8)	0.0006 (8)
C8	0.0400 (11)	0.0465 (11)	0.0350 (10)	0.0017 (9)	0.0010 (8)	0.0052 (8)
C9	0.0354 (10)	0.0480 (12)	0.0396 (10)	−0.0015 (9)	0.0060 (8)	−0.0007 (8)
C10	0.0486 (12)	0.0568 (13)	0.0318 (10)	−0.0047 (10)	0.0003 (8)	−0.0014 (9)
C11	0.0475 (12)	0.0549 (13)	0.0319 (10)	−0.0025 (10)	−0.0007 (8)	0.0056 (9)
C12	0.0462 (12)	0.0478 (12)	0.0528 (13)	−0.0038 (10)	0.0064 (10)	−0.0031 (10)
C13	0.0343 (9)	0.0441 (10)	0.0260 (9)	−0.0002 (8)	0.0015 (7)	−0.0005 (7)
C14	0.0398 (11)	0.0451 (11)	0.0349 (10)	0.0007 (9)	0.0005 (8)	−0.0049 (8)
C15	0.0483 (12)	0.0402 (11)	0.0445 (11)	0.0016 (9)	0.0048 (9)	−0.0016 (8)
C16	0.0470 (12)	0.0460 (11)	0.0352 (10)	0.0058 (9)	0.0049 (8)	0.0051 (8)
C17	0.0474 (11)	0.0478 (11)	0.0284 (9)	0.0054 (9)	−0.0029 (8)	0.0010 (8)
O2	0.0535 (8)	0.0405 (7)	0.0211 (6)	−0.0012 (6)	−0.0019 (5)	−0.0009 (5)
N2	0.0402 (9)	0.0382 (8)	0.0221 (7)	−0.0011 (7)	0.0007 (6)	−0.0018 (6)
C18	0.0361 (9)	0.0384 (10)	0.0226 (8)	−0.0007 (8)	0.0028 (7)	−0.0010 (7)
C19	0.0392 (10)	0.0357 (10)	0.0222 (8)	−0.0007 (8)	0.0008 (7)	−0.0007 (7)
C20	0.0379 (10)	0.0372 (10)	0.0253 (8)	−0.0006 (8)	0.0001 (7)	−0.0003 (7)
C21	0.0374 (10)	0.0366 (10)	0.0249 (8)	0.0011 (8)	−0.0013 (7)	0.0039 (7)
C22	0.0354 (9)	0.0396 (10)	0.0256 (8)	0.0016 (8)	0.0004 (7)	0.0037 (7)
C23	0.0361 (9)	0.0350 (9)	0.0260 (8)	0.0002 (8)	0.0026 (7)	−0.0001 (7)
C24	0.0367 (10)	0.0383 (10)	0.0251 (8)	0.0004 (8)	−0.0009 (7)	0.0008 (7)
C25	0.0392 (10)	0.0366 (10)	0.0306 (9)	0.0029 (8)	0.0009 (7)	0.0029 (7)
C26	0.0359 (10)	0.0377 (10)	0.0322 (9)	−0.0022 (8)	0.0051 (7)	−0.0033 (7)
C27	0.0395 (10)	0.0432 (11)	0.0258 (8)	−0.0019 (8)	0.0011 (7)	−0.0045 (7)
C28	0.0391 (10)	0.0397 (10)	0.0246 (8)	0.0006 (8)	−0.0008 (7)	0.0023 (7)
C29	0.0457 (11)	0.0401 (11)	0.0435 (11)	−0.0023 (9)	0.0056 (9)	−0.0064 (8)
C30	0.0348 (9)	0.0359 (10)	0.0215 (8)	−0.0012 (7)	0.0018 (7)	−0.0014 (6)
C31	0.0407 (10)	0.0375 (10)	0.0256 (8)	−0.0021 (8)	0.0000 (7)	−0.0031 (7)
C32	0.0458 (11)	0.0354 (10)	0.0327 (9)	−0.0022 (8)	0.0024 (8)	−0.0047 (7)
C33	0.0438 (11)	0.0376 (10)	0.0309 (9)	0.0010 (8)	0.0031 (8)	0.0049 (7)
C34	0.0436 (11)	0.0404 (10)	0.0239 (8)	0.0029 (8)	−0.0001 (7)	0.0003 (7)

### (E)-5-(4-Methylphenyl)-1-(pyridin-2-yl)pent-2-en-4-yn-1-one (2) Geometric parameters (Å, º)

**Table:**

O1—C1	1.226 (2)	O2—C18	1.228 (2)
N1—C13	1.346 (2)	N2—C30	1.345 (2)
N1—C17	1.337 (2)	N2—C34	1.339 (2)
C1—C2	1.474 (3)	C18—C19	1.476 (2)
C1—C13	1.498 (3)	C18—C30	1.498 (2)
C2—H2	0.9500	C19—H19	0.9500
C2—C3	1.335 (3)	C19—C20	1.336 (3)
C3—H3	0.9500	C20—H20	0.9500
C3—C4	1.411 (3)	C20—C21	1.420 (3)
C4—C5	1.206 (3)	C21—C22	1.203 (3)
C5—C6	1.426 (3)	C22—C23	1.430 (3)
C6—C7	1.397 (3)	C23—C24	1.400 (2)
C6—C11	1.401 (3)	C23—C28	1.402 (2)
C7—H7	0.9500	C24—H24	0.9500
C7—C8	1.388 (3)	C24—C25	1.385 (3)
C8—H8	0.9500	C25—H25	0.9500
C8—C9	1.390 (3)	C25—C26	1.396 (3)
C9—C10	1.397 (3)	C26—C27	1.396 (3)
C9—C12	1.504 (3)	C26—C29	1.507 (3)
C10—H10	0.9500	C27—H27	0.9500
C10—C11	1.374 (3)	C27—C28	1.380 (3)
C11—H11	0.9500	C28—H28	0.9500
C12—H12A	0.9800	C29—H29A	0.9800
C12—H12B	0.9800	C29—H29B	0.9800
C12—H12C	0.9800	C29—H29C	0.9800
C13—C14	1.380 (3)	C30—C31	1.385 (2)
C14—H14	0.9500	C31—H31	0.9500
C14—C15	1.390 (3)	C31—C32	1.384 (3)
C15—H15	0.9500	C32—H32	0.9500
C15—C16	1.385 (3)	C32—C33	1.385 (3)
C16—H16	0.9500	C33—H33	0.9500
C16—C17	1.379 (3)	C33—C34	1.384 (3)
C17—H17	0.9500	C34—H34	0.9500
			
C17—N1—C13	116.36 (17)	C34—N2—C30	116.40 (16)
O1—C1—C2	121.82 (18)	O2—C18—C19	121.85 (16)
O1—C1—C13	119.30 (19)	O2—C18—C30	119.62 (16)
C2—C1—C13	118.88 (15)	C19—C18—C30	118.53 (14)
C1—C2—H2	120.0	C18—C19—H19	120.5
C3—C2—C1	119.97 (17)	C20—C19—C18	118.97 (16)
C3—C2—H2	120.0	C20—C19—H19	120.5
C2—C3—H3	116.7	C19—C20—H20	116.8
C2—C3—C4	126.59 (19)	C19—C20—C21	126.41 (17)
C4—C3—H3	116.7	C21—C20—H20	116.8
C5—C4—C3	174.8 (2)	C22—C21—C20	173.42 (19)
C4—C5—C6	178.1 (2)	C21—C22—C23	177.61 (18)
C7—C6—C5	121.09 (18)	C24—C23—C22	119.68 (16)
C7—C6—C11	118.38 (19)	C24—C23—C28	118.69 (17)
C11—C6—C5	120.53 (18)	C28—C23—C22	121.63 (16)
C6—C7—H7	119.9	C23—C24—H24	119.8
C8—C7—C6	120.28 (18)	C25—C24—C23	120.40 (16)
C8—C7—H7	119.9	C25—C24—H24	119.8
C7—C8—H8	119.3	C24—C25—H25	119.5
C7—C8—C9	121.42 (18)	C24—C25—C26	121.03 (17)
C9—C8—H8	119.3	C26—C25—H25	119.5
C8—C9—C10	117.8 (2)	C25—C26—C27	118.29 (17)
C8—C9—C12	121.87 (19)	C25—C26—C29	120.02 (17)
C10—C9—C12	120.29 (19)	C27—C26—C29	121.68 (17)
C9—C10—H10	119.3	C26—C27—H27	119.4
C11—C10—C9	121.41 (19)	C28—C27—C26	121.22 (17)
C11—C10—H10	119.3	C28—C27—H27	119.4
C6—C11—H11	119.7	C23—C28—H28	119.8
C10—C11—C6	120.67 (19)	C27—C28—C23	120.36 (17)
C10—C11—H11	119.7	C27—C28—H28	119.8
C9—C12—H12A	109.5	C26—C29—H29A	109.5
C9—C12—H12B	109.5	C26—C29—H29B	109.5
C9—C12—H12C	109.5	C26—C29—H29C	109.5
H12A—C12—H12B	109.5	H29A—C29—H29B	109.5
H12A—C12—H12C	109.5	H29A—C29—H29C	109.5
H12B—C12—H12C	109.5	H29B—C29—H29C	109.5
N1—C13—C1	117.00 (17)	N2—C30—C18	116.97 (15)
N1—C13—C14	123.73 (18)	N2—C30—C31	123.55 (16)
C14—C13—C1	119.26 (16)	C31—C30—C18	119.47 (15)
C13—C14—H14	120.7	C30—C31—H31	120.5
C13—C14—C15	118.67 (18)	C32—C31—C30	119.03 (17)
C15—C14—H14	120.7	C32—C31—H31	120.5
C14—C15—H15	120.8	C31—C32—H32	120.9
C16—C15—C14	118.4 (2)	C31—C32—C33	118.19 (17)
C16—C15—H15	120.8	C33—C32—H32	120.9
C15—C16—H16	120.7	C32—C33—H33	120.6
C17—C16—C15	118.59 (19)	C34—C33—C32	118.83 (17)
C17—C16—H16	120.7	C34—C33—H33	120.6
N1—C17—C16	124.19 (18)	N2—C34—C33	123.94 (16)
N1—C17—H17	117.9	N2—C34—H34	118.0
C16—C17—H17	117.9	C33—C34—H34	118.0

### (E)-5-(4-Methylphenyl)-1-(pyridin-2-yl)pent-2-en-4-yn-1-one (2) Hydrogen-bond geometry (Å, º)

**Table:**

*D*—H···*A*	*D*—H	H···*A*	*D*···*A*	*D*—H···*A*
C2—H2···N1	0.95	2.52	2.832 (3)	100
C16—H16···N2^i^	0.95	2.66	3.555 (3)	158
C20—H20···N2^i^	0.95	2.71	3.465 (3)	136
C3—H3···O1^ii^	0.95	2.43	3.206 (3)	139
C19—H19···O2^iii^	0.95	2.57	3.379 (2)	143
C25—H25···O2^iv^	0.95	2.65	3.561 (2)	161

Symmetry codes: (i) *x*, −*y*+1/2, *z*+1/2; (ii) −*x*, −*y*+1, −*z*; (iii) *x*, −*y*+1/2, *z*−1/2; (iv) −*x*+1, −*y*, −*z*+1.
